# Changes in estrone and estradiol levels during follicle development: a retrospective large-scale study

**DOI:** 10.1186/s12958-015-0051-y

**Published:** 2015-06-02

**Authors:** Tomoya Segawa, Shokichi Teramoto, Kenji Omi, Osamu Miyauchi, Yoshiaki Watanabe, Hisao Osada

**Affiliations:** Shinbashi YUME Clinic, Excel Shinbashi, 2-5-1, Shinbashi, Minato-ku, Tokyo 105-0004 Japan

**Keywords:** Assisted reproductive technologies, Estradiol, Estrone, Follicular growth, Natural cycle IVF

## Abstract

**Background:**

The improved reagent for measuring estradiol (E2), the ST AIA-PACK iE2 reagent, has a higher specificity for the measurement of E2 levels than the original ST AIA-PACK E2 reagent, because of its lower cross-reactivity with estrone (E1). As we had E2 data obtained with either of the reagents, we analyzed changes in E1 and E2 levels during follicle development.

**Methods:**

The study included 14371 serum hormone measurements from 4412 patients who underwent oocyte retrieval or frozen/thawed embryo transfer in natural cycle *in vitro* fertilization in Shinbashi YUME clinic, Tokyo, between June 2011 and May 2014. The age of the patients ranged from 24 to 48 year (mean and standard deviation, 39.8 ± 4.0 year). Patients were categorized into three age groups (<38 year, 38–40 year, and >40 year) and into 10 groups of largest follicle diameter from 11 to 20 mm, with 1-mm intervals. Serum E2 levels were measured in the follicular phase with either the ST AIA-PACK E2 reagent or the ST AIA-PACK iE2 reagent, and the data were compared. Also, for 26 randomly selected samples, E2 was measured using both reagents, together with E1 and E3, and the E1/E2 ratios were compared.

**Results:**

E2 concentrations measured with the ST AIA-PACK iE2 reagent were significantly lower than those measured with the ST AIA-PACK E2 reagent in the largest follicle diameter category of 11–17 mm in the <38 year group, in the largest follicle diameter category of 11–18 mm in the 38–40 year group, and in the largest follicle diameter category of 11–15 mm in the >40 year group. The serum E1/E2 ratio in the 26 samples was 3.4 ± 1.1 and 0.7 ± 0.1 in the early follicular phase and in the ovulatory phase, respectively.

**Conclusions:**

The difference between the E2 concentrations measured with the ST AIA-PACK E2 reagent and the ST AIA-PACK iE2 reagent tended to decrease as the follicle diameter increased, particularly in the older patients, which suggests E1 secretion is more abundant in the early follicular phase and in younger patients than in the ovulatory phase and in older patients.

## Background

The serum estradiol (E2) concentration is used to evaluate follicle maturation. This measurement is essential to predict the timing of ovulation when treating infertility patients. As the date of ovulation approaches, the E2 level changes dynamically day-by-day, particularly in natural cycle *in vitro* fertilization (IVF). In pre-menopausal women, E2 is mainly secreted by granulosa cells in the follicles. As these cells divide and proliferate within a follicle, increasing in number as the follicle grows, the E2 level also increases. Thus, the E2 concentration is a good index of follicular maturation. Menopausal and post-menopausal women, as well as obese women, have elevated serum levels of estrone (E1), and E1 is also secreted by tissues other than the ovary [1–6]. However, the action and role of E1 and its secretion dynamics are still not well understood.

In patients undergoing infertility treatment using assisted reproductive technologies in our clinic, the E2 level is measured using an enzyme immunoassay analyzer (AIA-1800 or AIA-2000; TOSOH Corporation, Tokyo, Japan). The E2 levels are measured in real time using a competitive enzyme immunoassay based on the fluorescence enzyme immunoassay (FEIA) method. It is performed entirely within a supplied test cup (ST AIA-PACK E2 test kit or ST AIA-PACK iE2 test kit). E2 present in the test sample competes with alkaline phosphatase-labeled E2 in the cup for a limited number of binding sites on E2-specific antibodies immobilized on magnetic beads. The beads are washed to remove any unbound enzyme-labeled E2 and are then incubated with a fluorogenic substrate. The E2 concentration in the sample is calculated based on the time the enzyme takes to produce a fluorescent substance. Serum E2 levels are measured in approximately 20 min. Initially, we used the ST AIA-PACK E2 test kit (hereinafter referred to as “the ST AIA-PACK E2 reagent”), but in August 2012, we changed to the ST AIA-PACK iE2 test kit (hereinafter referred to as the “ST AIA-PACK iE2 reagent). The ST AIA-PACK iE2 reagent uses CRM577, a reference material certified by the Institute for Reference Materials and Measurements, for calibration. Results with the ST AIA-PACK iE2 reagent correlate well with results of isotope dilution-gas chromatography/mass spectrometry. Additionally, the ST AIA-PACK iE2 reagent uses an antibody with a higher E2 specificity than the ST AIA-PACK E2 reagent, which uses a polyclonal antibody generated by immunizing a rabbit with antigen and purifying its blood. For the ST AIA-PACK iE2 reagent, the antibody is obtained by exenterating the spleen of an antigen-immunized rabbit, isolating the B cells, and creating hybridoma cells by fusing the B cells with myeloma cells; these hybridoma cells can divide indefinitely, and the cells that generate an antibody with a higher E2 specificity are then selected. Table 1 shows the cross-reactivity of both reagents. The ST AIA-PACK iE2 reagent is shown to be more specific than the ST AIA-PACK E2 reagent as it has a much lower cross-reactivity with E1 and estriol (E3). In other words, E1 and E3 secretion is more likely to be included in the serum E2 concentration measured with the ST AIA-PACK E2 reagent than in that measured with the ST AIA-PACK iE2 reagent.

**Table 1:** Cross-reactivity of ST AIA-PACK E2 and ST AIA-PACK iE2 reagents

	Cross-reactivity (mol %)	
Substance	ST AIA-PACK E2 reagent	ST AIA-PACK iE2 reagent
Estradiol (E2)	100	100
Estrone (E1)	7.2	1.1
Estriol (E3)	4.9	0.25

Please refer to the following websites for additional data. (The text is in Japanese but the data referred to above is in English)

http://www.info.pmda.go.jp/downfiles/ivd/PDF/480201_13A2X00174000017_B_02_01.pdf

http://www.info.pmda.go.jp/downfiles/ivd/PDF/480201_13A2X00174000006_A_04_01.pdf

We hypothesized that a comparison of the measurements obtained by the two different reagents would allow us to indirectly evaluate the serum levels of hormones other than E2 with which the ST AIA-PACK E2 reagent had high cross-reactivity. We carried out a statistical analysis and compared the E2 measurements obtained with the ST AIA-PACK E2 reagent and ST AIA-PACK iE2 reagent according to follicle size in a natural cycle IVF setting. Changes in the E2 levels observed during the course of infertility treatment are reported. We also describe cases in which the different cross-reactivity to E1 and E3 between the two reagents was reflected in the difference in the measured concentrations.

## Methods

A total of 14371 serum hormone data measurements from 4412 women who underwent oocyte retrieval or frozen/thawed embryo transfer in natural cycle IVF in our clinic between June 2011 and May 2014 were included in this study. These patients were treated with IVF or intracytoplasmic sperm injection (ICSI) because of female-related (12.1 %), male-related (28.0 %), or unexplained (59.8 %) issues. The patients’ age ranged from 24 to 48 year (mean age and standard deviation, 39.8 ± 4.0 year). The average body mass index was 20.7 ± 2.6 (range: 14.8–41.0). Most patients were Japanese (99.2 %), followed by other Asians (0.7 %) and other ethnic groups (0.1 %). Samples were taken from the patients between day 8 and day 20 of their menstrual cycle, before the onset of the luteinizing hormone (LH) surge, i.e., when the LH level was <20 IU/L and the progesterone (P4) level was <1.0 ng/mL. Cases were excluded if either of the following criteria were met: 1) more than two follicles ≥11 mm in diameter were observed on ultrasonography and 2) the diameter of the largest follicle was ≥21 mm. Approval of this study was obtained from the independent Institutional Review Board of Shinbashi YUME Clinic (SYC2014-8, approved 1 July, 2014). All patients received detailed information about the proposed treatment. Written informed consent was obtained from all participants; they also agreed that their de-identified data could be used for research purposes.

Serum concentrations of E2, LH, follicle-stimulating hormone (FSH), and P4 were measured in all patients on the AIA-1800 or AIA-2000 analyzer using the FEIA method. Follicle size during the natural cycle, with no administration of ovulation-inducing drugs, was determined by ultrasound examination using the HI VISION AVIUS system (Hitachi Medical Corporation, Tokyo, Japan). Follicle maturation was considered complete when serum E2 levels were ≥200 pg/mL and the diameter of the largest follicle was ≥18 mm.

When the follicles reached maturation, a gonadotropin-releasing hormone (GnRH) agonist (Suprecur, Mochida Pharmaceutical Co., LTD., Tokyo, Japan) was administered to trigger the endogenous LH surge, and oocytes were retrieved 34–36 h later. The retrieved oocytes were inseminated by conventional methods or ICSI. They were cultured until they reached the expanded blastocyst stage, and then transferred as a fresh embryo or vitrified for a later frozen/thawed transfer. For the frozen/thawed transfer carried out in the natural cycle, serum hormone measurement and the ultrasound examination were performed as in the oocyte retrieval cycle; the embryo was thawed 4–5 days after ovulation, and the blastocyst was transferred. Oocyte retrieval, embryo transfer, and embryo culture were all carried out following the methods stipulated by Teramoto *et al.* [7] and Kato *et al.* [8, 9].

Serum E2 concentrations for the 14371 samples obtained during the oocyte retrieval cycles and frozen/thawed embryo transfer cycles, measured with the ST AIA-PACK E2 reagent or ST AIA-PACK iE2 reagent, were categorized by the largest follicle diameter (10 groups, from 11 to 20 mm, with 1-mm intervals) and by age (<38 year, 38–40 year, and >40 year). Since most of the patients in our clinic have histories of failed IVF treatment in other institutions, our patient population tends to be much older than that of other institutions. For this reason, patients in the <38 year, 38–40 year, and >40 year categories were considered young, older, and old, respectively. In total, 4032 measurements were in the <38 year group (1256 and 2776 with the ST AIA-PACK E2 reagent and ST AIA-PACK iE2 reagent, respectively), 3740 were in the 38–40 year group (1307 and 2433 with the ST AIA-PACK E2 reagent and ST AIA-PACK iE2 reagent, respectively), and 6599 were in the >40 year group (2720 and 3879 with the ST AIA-PACK E2 reagent and ST AIA-PACK iE2 reagent, respectively). The serum E2 levels measured with each reagent were then compared within each follicle size category. Similarly, the serum E2 concentration and serum FSH concentration were compared between the three age groups in each follicle size category. Furthermore, serum E2 (measured with both reagents), serum E1, and serum E3 concentrations (measured by the radioimmunoassay method; BML Inc., Tokyo, Japan) were measured in 12 randomly selected cases in the early follicular phase and in 14 randomly selected cases in the ovulatory phase, and the measurements in the two phases were compared. All data were analyzed using the *t*-test by JMP 11.2 (SAS Institute, Cary, NC, USA). *P* < 0.01 was considered to indicate statistical significance.

## Results

Table 2 shows the comparison between the serum E2 concentrations measured with the two reagents. The data are categorized by age and largest follicle diameter. In the <38 year group, E2 concentrations measured with the ST AIA-PACK iE2 reagent were significantly lower than those measured with the ST AIA-PACK E2 reagent in the largest follicle diameter categories ranging from 11 to 17 mm. However, in the largest follicle diameter categories from 18 to 20 mm, the E2 concentrations measured using the two reagents did not differ. In the 38–40 year group, the E2 concentrations measured with the ST AIA-PACK iE2 reagent were significantly lower than those measured with the ST AIA-PACK E2 reagent in the largest follicle diameter categories ranging from 11 to 18 mm, but no significant difference was noted for the categories from 19 to 20 mm. In the >40 year group, the E2 concentrations measured with the ST AIA-PACK iE2 reagent were significantly lower than those measured with the ST AIA-PACK E2 reagent in the largest follicle diameter categories ranging from 11 to 15 mm, but no significant difference was noted for the categories from 16 to 20 mm. Table 3 shows the comparison of serum E2 concentrations (measured with the ST AIA-PACK iE2 reagent) and serum FSH concentrations between the age groups for each largest follicle diameter category. There were four patterns of significant differences between the three age groups. When focusing on the <38 year and the >40 year groups, serum E2 concentrations were significantly higher in the older age group than in the younger age group for the 11 mm to 18 mm largest follicle diameter categories, but there was no significant difference between the groups for the 19 mm and 20 mm categories. Serum FSH concentrations were significantly higher in the older age group for all largest follicle diameter categories. Table 4 shows the comparison of the mean serum estrogen concentrations (E1, E2, and E3) in 12 randomly selected cases from the early follicular phase and 14 randomly selected cases from the ovulatory phase. The ratios of serum E2 concentrations measured with the ST AIA-PACK iE2 reagent to those measured with the ST AIA-PACK E2 reagent were 2.9 ± 0.6 and 0.9 ± 0.1 for the early follicular phase group and the ovulatory phase group, respectively (*P* < 0.01). Furthermore, the serum E1 concentration to serum E2 concentration ratios measured with the ST AIA-PACK iE2 reagent (E1/E2) were 3.4 ± 1.1 and 0.7 ± 0.1 in the early follicular phase group and the ovulatory phase group, respectively (*P* < 0.01). Serum E3 concentrations were below the detection range in both groups.

**Table 2:** E2 concentrations measured with the E2 reagent and iE2 reagent according to largest follicle size

Diameter of the largest follicle	Serum E2 (pg/mL)					
	Age <38 year (*n* = 4032)		Age 38–40 year (*n* = 3740)		Age >40 year (*n* = 6599)	
11 mm	E2 reagent (*n* = 125)	99.9 ± 32.6^a^	E2 reagent (*n* = 99)	101.5 ± 30.9^a^	E2 reagent (*n* = 195)	113.4 ± 45.3^a^
	iE2 reagent (*n* = 191)	68.0 ± 34.7^a^	iE2 reagent (*n* = 147)	71.0 ± 40.4^a^	iE2 reagent (*n* = 220)	86.8 ± 51.8^a^
12 mm	E2 reagent (*n* = 114)	105.8 ± 35.6^a^	E2 reagent (*n* = 122)	113.5 ± 40.9^a^	E2 reagent (*n* = 226)	125.3 ± 48.2^a^
	iE2 reagent (*n* = 234)	80.4 ± 41.8^a^	iE2 reagent (*n* = 177)	84.7 ± 42.9^a^	iE2 reagent (*n* = 297)	102.8 ± 53.5^a^
13 mm	E2 reagent (*n* = 127)	122.2 ± 44.6^a^	E2 reagent (*n* = 114)	135.9 ± 49.0^a^	E2 reagent (*n* = 242)	142.2 ± 49.1^a^
	iE2 reagent (*n* = 241)	87.3 ± 42.6^a^	iE2 reagent (*n* = 200)	96.7 ± 44.6^a^	iE2 reagent (*n* = 284)	116.1 ± 61.2^a^
14 mm	E2 reagent (*n* = 125)	130.7 ± 38.8^a^	E2 reagent (*n* = 125)	155.2 ± 57.4^a^	E2 reagent (*n* = 234)	166.6 ± 59.8^a^
	iE2 reagent (*n* = 246)	100.3 ± 51.7^a^	iE2 reagent (*n* = 182)	115.8 ± 58.6^a^	iE2 reagent (*n* = 313)	126.1 ± 64.0^a^
15 mm	E2 reagent (*n* = 118)	168.0 ± 57.0^a^	E2 reagent (*n* = 154)	168.8 ± 60.2^a^	E2 reagent (*n* = 313)	187.0 ± 70.6^a^
	iE2 reagent (*n* = 268)	132.6 ± 64.2^a^	iE2 reagent (*n* = 238)	150.2 ± 66.5^a^	iE2 reagent (*n* = 407)	170.6 ± 76.5^a^
16 mm	E2 reagent (*n* = 134)	186.7 ± 67.8^a^	E2 reagent (*n* = 173)	195.4 ± 60.0^a^	E2 reagent (*n* = 351)	210.4 ± 64.6
	iE2 reagent (*n* = 287)	160.0 ± 77.9^a^	iE2 reagent (*n* = 222)	177.0 ± 78.2^a^	iE2 reagent (*n* = 444)	197.4 ± 79.5
17 mm	E2 reagent (*n* = 151)	228.5 ± 66.2^a^	E2 reagent (*n* = 158)	236.6 ± 64.0^a^	E2 reagent (*n* = 351)	231.8 ± 61.1
	iE2 reagent (*n* = 322)	200.2 ± 85.7^a^	iE2 reagent (*n* = 282)	216.5 ± 78.5^a^	iE2 reagent (*n* = 489)	222.3 ± 86.6
18 mm	E2 reagent (*n* = 151)	241.0 ± 75.0	E2 reagent (*n* = 143)	257.3 ± 60.9^a^	E2 reagent (*n* = 348)	255.9 ± 59.2
	iE2 reagent (*n* = 323)	229.2 ± 85.4	iE2 reagent (*n* = 356)	233.5 ± 88.9^a^	iE2 reagent (*n* = 529)	247.0 ± 82.2
19 mm	E2 reagent (*n* = 102)	253.4 ± 66.1	E2 reagent (*n* = 112)	260.8 ± 68.2	E2 reagent (*n* = 230)	259.2 ± 64.6
	iE2 reagent (*n* = 242)	249.6 ± 78.0	iE2 reagent (*n* = 273)	256.0 ± 79.5	iE2 reagent (*n* = 368)	261.0 ± 83.5
20 mm	E2 reagent (*n* = 109)	275.2 ± 72.2	E2 reagent (*n* = 107)	279.9 ± 75.1	E2 reagent (*n* = 230)	281.5 ± 74.2
	iE2 reagent (*n* = 422)	275.6 ± 95.2	iE2 reagent (*n* = 356)	285.1 ± 88.0	iE2 reagent (*n* = 528)	280.6 ± 92.0

Data are presented as mean ± standard deviation

^a^Significant differences in serum E2 concentration were found between measurements performed using the ST AIA-PACK E2 reagent and the ST AIA-PACK iE2 reagent (*P* <0.01)

*E2* estradiol, *E2 reagent* ST AIA-PACK E2 reagent, *iE2 reagent* ST AIA-PACK iE2 reagent

**Table 3:** E2 and FSH concentrations in the three age groups, classified according to follicle size

Diameter of the largest follicle	Serum E2 (pg/mL) measured by iE2 reagent			Serum FSH (mIU/mL)		
	(*n* = 9088)			(*n* = 9088)		
	Age <38 year	Age 38–40 year	Age >40 year	Age <38 year	Age 38–40 year	Age >40 year
11 mm	68.0 ± 34.7^a^	71.0 ± 40.4^a^	86.8 ± 51.8^a^	8.6 ± 2.4^b^	9.5 ± 4.6	10.9 ± 6.8^b^
12 mm	80.4 ± 41.8^a^	84.7 ± 42.9^a^	102.8 ± 53.5^a^	8.4 ± 2.4^a^	8.8 ± 3.0^a^	11.1 ± 7.1^a^
13 mm	87.3 ± 42.6^a^	96.7 ± 44.6^a^	116.1 ± 61.2^a^	7.9 ± 2.3^a^	8.3 ± 3.2^a^	10.3 ± 7.0^a^
14 mm	100.3 ± 51.7^c^	115.8 ± 58.6^c^	126.1 ± 64.0^c^	7.4 ± 1.9^c^	8.5 ± 3.5^c^	9.9 ± 8.5^c^
15 mm	132.6 ± 64.2^d^	150.2 ± 66.5^d^	170.6 ± 76.5^d^	7.3 ± 2.3^c^	8.3 ± 3.7^c^	8.8 ± 4.3^c^
16 mm	160.0 ± 77.9^a^	177.0 ± 78.2^a^	197.4 ± 79.5^a^	7.1 ± 2.5^a^	7.7 ± 3.6^a^	9.0 ± 5.2^a^
17 mm	200.2 ± 85.7^b^	216.5 ± 78.5	222.3 ± 86.6^b^	6.8 ± 2.2^d^	7.7 ± 3.1^d^	8.8 ± 6.0^d^
18 mm	229.2 ± 85.4^b^	233.5 ± 88.9	247.0 ± 82.2^b^	6.6 ± 2.3^d^	7.2 ± 2.8^d^	8.5 ± 5.1^d^
19 mm	249.6 ± 78.0	256.0 ± 79.5	261.0 ± 83.5	6.3 ± 2.3^a^	6.8 ± 2.3^a^	8.3 ± 6.4^a^
20 mm	275.6 ± 95.2	285.1 ± 88.0	280.6 ± 92.0	6.5 ± 2.2^a^	6.8 ± 2.3^a^	7.8 ± 4.0^a^

Data are presented as mean ± standard deviation

^a^Significant differences were found between the <38 year group and the >40 year group, as well as between the 38–40 year group and the >40 year group (*P* < 0.01)

^b^Significant differences were found between the <38 year group and the >40 year group (*P* < 0.01)

^c^Significant differences were found between the <38 year group and the 38–40 year group, as well as between the <38 year group and the >40 year group (*P* < 0.01)

^d^Significant differences were found among all three age groups (*P* < 0.01)

*E2* estradiol, *FSH* follicle-stimulating hormone, *iE2 reagent* ST AIA-PACK iE2 reagent

**Table 4:** Comparison of estrogen measurements in the early follicular phase and the ovulatory phase

	Early follicular phase	Ovulatory phase
	(*n* = 12)	(*n* = 14)
Serum E2 (pg/mL)		
Measured with ST AIA-PACK E2 reagent (A)	58.7 ± 7.9	245.4 ± 50.5
Measured with ST AIA-PACK iE2 reagent (B)	21.3 ± 4.7	262.9 ± 54.2
(A)/(B)	2.9 ± 0.6^a^	0.9 ± 0.1^a^
Serum E1 (pg/mL)	68.0 ± 13.9	191.1 ± 51.9
E1/E2 (measured with A)	3.4 ± 1.1^a^	0.7 ± 0.1^a^
Serum E3 (pg/mL)^b^	<5.0	<5.0

Data are presented as mean ± standard deviation

^a^Significant differences were found between the early follicular phase and the ovulatory phase groups (*P* <0.01)

^b^Serum E3 concentrations were below the detection range for both groups

*E1* estrone, *E2* estradiol, *E3* estriol

## Discussion

In the present study, we analyzed the differences in estrogen concentration measurements according to the reagent used, considering the different properties of the two reagents used for measuring serum E2 concentrations. The improved specificity of the new E2 measuring reagent from TOSOH Corp. is attributable to the use of a monoclonal antibody rather than a polyclonal antibody. Therefore, we presumed that the content of estrogen besides E2 (mainly E1) could be measured indirectly by analyzing the differences in measurements obtained using the ST AIA-PACK E2 reagent and the ST AIA-PACK iE2 reagent. We could not measure E2 in all the 14371 samples using both the ST AIA-PACK E2 reagent and the AIA-PACK iE2 reagent, although this would have been ideal, because this would have been very expensive. Instead, we measured E2 (using both reagents), E1, and E3 at the same time in a few samples, analyzed the differences, and compared the E1 to E2 ratios. The E1/E2 ratio was high in the samples taken from the early follicular phase and there was a significant difference between the concentrations measured with the ST AIA-PACK E2 reagent and ST AIA-PACK iE2 reagent, suggesting that the concentration of E1 was high in these samples. In samples taken during the ovulatory phase, the E1/E2 ratio was small and there was only a small difference in the concentrations measured with the ST AIA-PACK E2 reagent and ST AIA-PACK iE2 reagent, indicating a lower concentration of E1 in these samples.

The LH surge is triggered by positive feedback from the hypothalamic-pituitary axis, by the increase in estrogen levels in the late follicular phase [10]. If a decrease in serum E1 causes the E2/E1 ratio to increase in the ovulatory phase (follicle diameter, 19–20 mm), as seen in the current study, this phenomenon may trigger the onset of the LH surge. Conversely, the onset of the LH surge may be suppressed when E1 secretion is high (follicle diameter, ≤18 mm). The possible role of E1 secretion in the mid-to-late follicular phase in maintaining follicle development by suppressing the LH surge until the follicle is fully matured is extremely interesting. In the >40 year group however, the difference in the serum E2 measurements observed between the two reagents disappeared after the mid-follicular phase (follicle diameter, 16 mm) and a relative increase in E2 (or decrease in E1) was observed. In older infertility patients, premature elevation of serum LH levels is often observed, before the follicle is fully developed and the premature LH surge arrests follicular growth. In these situations, good mature oocytes cannot be obtained. Aging is known to affect pulsatile GnRH secretion and the pituitary reaction [11]. In the present study, E1 decrease occurred earlier in the >40 year group than in the younger age groups. In the ≤16 mm follicle diameter category, the serum E2 levels were significantly higher in the >40 year group than in the younger age groups (Table 3). Several reports describe an increase in the serum E2 concentration in the follicular phase of older women [12–15]. A decrease in serum inhibin B in older women causes an increase in FSH secretion through negative feedback, which in turn stimulates the granulosa cells in the ovary, resulting in an elevated serum E2 level [12, 14, 15]. Welt *et al.* also showed that the androstenedione/E1 ratio decreased in older women in the early-to-mid follicular phase [16]. Androstenedione is converted to E1 by the enzymatic activity of aromatase [17], which also converts testosterone into E2. Therefore, aromatase activity in the ovary may increase in older women. In this study, there was no difference in the E2 levels measured with the ST AIA-PACK E2 reagent and ST AIA-PACK iE2 reagent in the follicle diameter categories from 16 to 20 mm in the >40 year group, suggesting that E1 levels had decreased (Table 2).

There are many reports on the association of E1 with estrogen-dependent breast cancer tumor growth and coronary artery disease [18–20]. The bioactivity of E1 is considered weaker than that of E2 [21]. Kushnir *et al.* examined the E1 and E2 concentrations in follicular fluid (FF) in the early follicular phase and at oocyte retrieval and found a more than 10-fold higher E1/E2 ratio in the early follicular phase than at oocyte retrieval. They also showed that androstenedione concentrations in the early follicular phase were approximately 60 times greater than those measured at oocyte retrieval [22]. Costa *et al.* measured hormone concentrations in the FF at oocyte retrieval, and compared FF in the follicles containing a mature and an immature oocyte. The E2 and testosterone levels were significantly lower in the FF containing mature oocytes than in the FF containing immature oocytes, but there was no significant difference in E1 and progesterone levels between the two kinds of FF [23]. McNatty *et al.* examined hormone concentrations in the FF of follicles ≥8 mm in diameter and found that E1/E2 ratio was similar in the early, mid, and late follicular phases, but the androstenedione/E1 ratio decreased as the follicular phase progressed from the early, mid, to late phase. They also found that the E1/E2 ratio was lower in blood samples taken from the veins in the ovary containing a follicle ≥8 mm than in samples taken from the veins in the ovary not containing a follicle ≥8 mm [24]. While there are several reports on the E1 concentration in the FF, the role of E1 in follicle development has not been reported. Although the present study is an indirect data analysis, the shift in the secretion level of estrogen (E2 and E1) is of great significance. The E1/E2 ratio, in addition to the change in follicle size, might play an important role in follicle development and maturation and ovulation of the oocyte.

## Conclusions

The present study revealed a shift in estrogen secretion during follicle development. In general, fertility centers use ovulation-inducing drugs to have multiple follicles develop, thus making it impossible to measure the hormonal concentrations per single dominant follicle. We, on the other hand, treat patients with the natural cycle protocol, which does not require the use of ovulation-inducing drugs. The timing of ovulation was determined by measuring the diameter of the developing dominant follicle by ultrasonography and the serum E2 concentrations each time the patient visited the clinic. Therefore, we could accumulate a large amount of data, and we believe this kind of data is very valuable. We also found that the difference between the E2 concentrations measured with the ST AIA-PACK E2 reagent and the ST AIA-PACK iE2 reagent tended to decrease as the follicle diameter increased, particularly in the older patients, which suggests that E1 is more abundant in the early follicular phase and in younger patients than in the ovulatory phase and in older patients. We are planning to extend the scope of the study and examine various aspects such as the relationship between the level of estrogen and oocyte quality, and their influence on pregnancy.

## Abbreviations

E1: Estrone
E2: Estradiol
E3: Estriol
FEIA: Fluorescence enzyme immunoassay
FF: Follicular fluid
FSH: Follicle-stimulating hormone
GnRH: Gonadotropin-releasing hormone
ICSI: Intracytoplasmic sperm injection
IVF: *In vitro* fertilization
LH: Luteinizing hormone
P4: Progesterone
